# Review of Diagnostic and Therapeutic Approach to Canine Myxomatous Mitral Valve Disease

**DOI:** 10.3390/vetsci4040047

**Published:** 2017-09-26

**Authors:** Giulio Menciotti, Michele Borgarelli

**Affiliations:** Department of Small Animal Clinical Sciences, Virginia-Maryland College of Veterinary Medicine, 205 Duck Pond Dr., Blacksburg, VA 24061, USA; micheb1@vt.edu

**Keywords:** dogs, heart, echocardiography, mitral repair

## Abstract

The most common heart disease that affects dogs is myxomatous mitral valve disease. In this article, we review the current diagnostic and therapeutic approaches to this disease, and we also present some of the latest technological advancements in this field.

## 1. Introduction

Among canine cardiac diseases, myxomatous mitral valve disease (MMVD) represents, by far, the most common. In general, the disease is more prevalent in small breeds than in large breeds, and some small breeds are reported to have an incidence close to 100% over a dog’s lifetime [1,2]. However, large breed dogs can be affected as well [3,4].

A heritable, genetically-determined component for the disease can be implied by the strong predilection for small breeds in general, and particularly for certain breeds (i.e., Cavalier King Charles Spaniels, Dachshund), in which the heritability of disease status and severity has been demonstrated [5,6,7]. However, the etiology of the myxomatous process is still unknown and under investigation. Some experimental evidence seems to support a possible role of the serotonin signaling pathway triggered by altered mechanical stimuli in the disease development [8,9,10,11,12]. In this hypothesis, the activation of tension mechanosensors is associated with a local increase of expression of the main serotonin synthesizing enzyme, tryptophan hydroxylase 1 (TPH1) and, therefore, a postulated increase in autocrine production of serotonin at the level of the leaflets [9,13,14,15]. Also supporting this hypothesis, in vitro experiments of Lacerda and colleagues showed that canine mitral leaflets undergoing static and cyclic strains, compared to unstrained controls, express increased amount of activated interstitial cell phenotype markers, glycosaminoglycans synthetic enzymes, proteoglycans, extracellular matrix catabolic enzymes, and TPH1 [14]; however, a pig model of MR failed to show increased mitral valve (MV) expression of TPH1 compared to controls [9]. Differences in species and techniques used do not allow a direct comparison of the two studies, and further investigation of this pathway is needed for confirming this hypothesis.

The pathophysiology of the disease, on the other hand, is well described. Valves affected by the myxomatous degeneration undergo a process of microscopic structural derangement which ultimately leads to the macroscopic alterations that cause incomplete apposition of the mitral leaflets and, therefore, mitral regurgitation (MR). In valves affected by MMVD, cellularity and matrix deposition are increased [8,12,15]. The valvular interstitial cells (VIC) change phenotype, transforming into a more myofibroblast type [16]; collagenolytic enzymes and matrix metalloproteinases are increasingly expressed [14], and glycosaminoglycans infiltrate the *spongiosa* layer, disrupting collagen fiber orientation. These changes impair the biomechanical properties of the valve, and its ability to withstand the forces to which is subjected [17]. Grossly, affected MVs appear thick and nodular, and the degree of severity and distribution of the lesions is age related [18]. Lesions usually start as isolated nodules on the free edge of the leaflets, and they progress to eventually coalesce and involve larger areas of the valve and of the chordae tendineae, therefore compromising leaflet coaptation and causing MR. The severity of MR ultimately determines the amount of left atrial and left ventricular remodeling and the hemodynamic consequences of the disease.

## 2. Diagnosis

**Figure 1:**
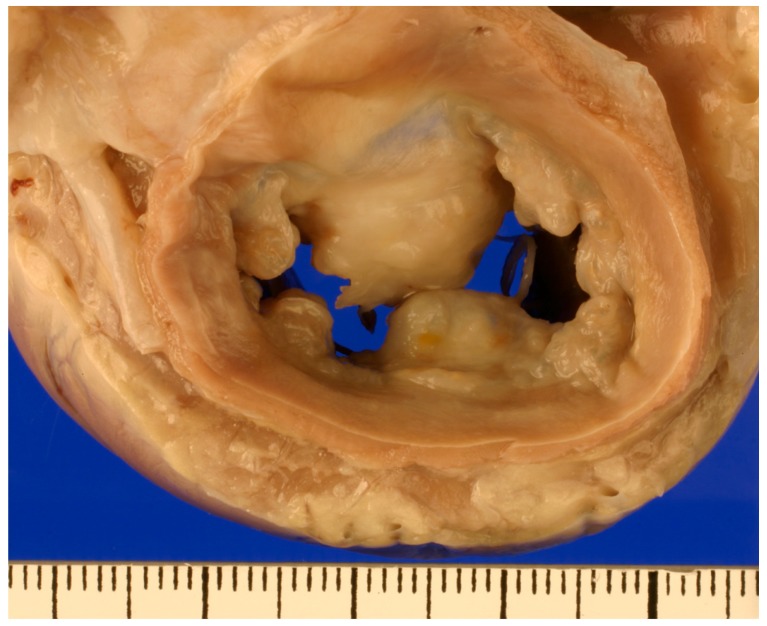
Gross pathology specimen fixed in 10% formalin showing the MV of a dog affected by MMVD. The LA was removed and thick, nodular mitral leaflets are shown.

Even though in geriatric dogs of predisposed breeds the identification of a left apical holosystolic murmur is almost unequivocally caused by MMVD [19], many dogs affected by mild disease can present without an audible murmur [20] and, therefore, the definitive diagnosis of this condition is made by performing an echocardiographic exam. Affected valves have a nodular, thickened appearance (Figure 1). The anterior mitral leaflet is more often affected, and bi-leaflet involvement is also common, while isolated lesions on the posterior leaflet are somewhat rare [21]. The free edge of the leaflet is usually more affected than the annular region.

Evidence of MR is confirmed by Doppler echocardiography that allows identifying the presence of a systolic, high velocity regurgitant jet originating from the left ventricle, accelerating at the level of the MV, and directed into the left atrium (LA). Given the diverse involvement of the two leaflets, often the regurgitant jet appears eccentric and multiple jets could be present.

## 3. Assessing Disease Severity

The severity of MMVD is assessed by combining information from history, physical exam and various imaging modalities. Exercise intolerance, presence of cough, decreased appetite, difficulty breathing, and syncopal episodes are history findings that have been associated with a poorer prognosis in affected dogs [22,23,24]. At physical examination, murmur grade and absence of sinus arrhythmia can be predictive of cardiac death [22]. Thoracic radiographs allow assessment of cardiac enlargement [25,26] and presence of pulmonary edema [27], which in dogs with MMVD is usually the hallmark of congestive heart failure (CHF). Echocardiography, however, should be considered the non-invasive gold standard for cardiac evaluation [28]. Therefore, the main echocardiographic parameters used for assessing MMVD are reviewed in the next section, followed by a brief outline of some most recent advances in echocardiography.

### 3.1. Conventional Echocardiography

#### 3.1.1. Assessing MR Severity

**Figure 2:**
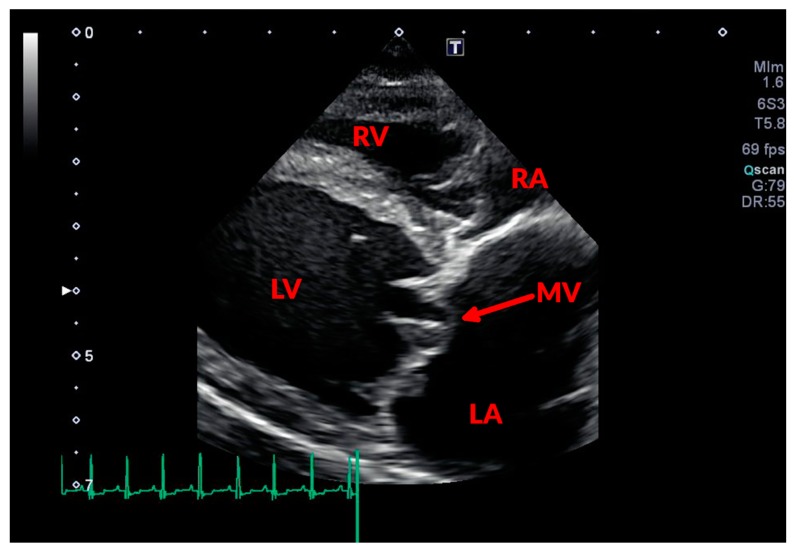
Two-dimensional echocardiographic image of a dog with mitral prolapse. In this right parasternal long axis four-chamber view, the mitral leaflets, thickened and nodular in appearance, can be noticed bulging into the LA. RA: right atrium; RV: right ventricle; LV: left ventricle; LA: left atrium; and MV: mitral valve.

**Figure 3:**
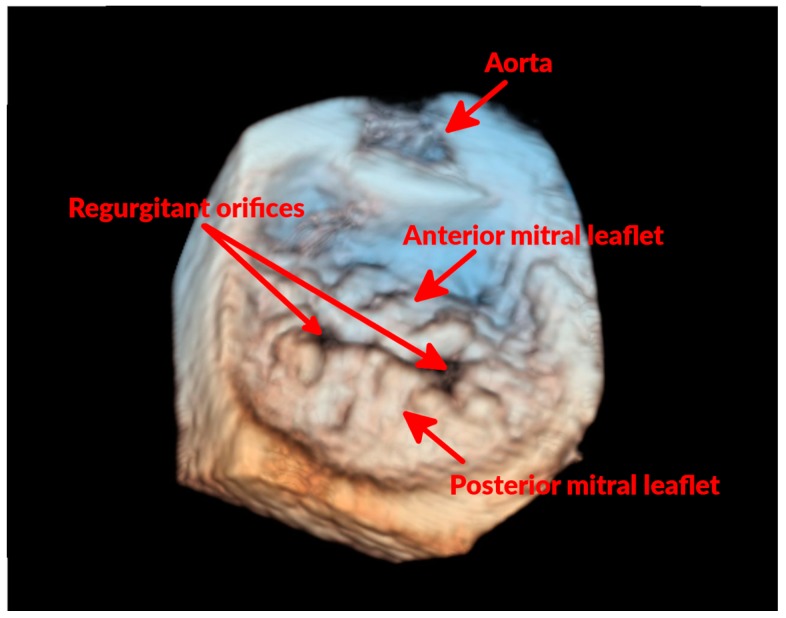
Three-dimensional echocardiographic “surgical” view of the MV of a dog with mitral prolapse. In this three-dimensional echocardiographic image, the MV is visualized as seen from the LA. It can be noticed how several areas of both anterior and posterior MV leaflets are bulging. It can also be noticed that the two leaflets fail to coapt in two areas (regurgitant orifices), which is where the MR occurs.

During systole, some portions of one or both leaflets could appear bulging into the LA as a result of mitral prolapse (Figure 2 and Figure 3). The chordae tendinae can be involved in the degenerative process as well, appearing thickened and elongated. Due to these changes, they fail in their mechanical task of holding the valve in place during systole, causing a portion of the valve to prolapse or even “flail” into the LA. Assessing MR severity is important for monitoring disease progression, and could help to predict the outcome of affected dogs [29,30], as MR represents the main hemodynamic derangement of the disease and, therefore, the cause of congestive heart failure (CHF). Assessment of the severity of MR can be semi-quantitative or quantitative. Probably the most commonly used semi-quantitative method is the ratio between the area of the regurgitant jet signal and the LA area (ARJ/LAA) [31]; with this technique MR is defined as mild (ARJ/LAA < 30%), moderate (30% ≤ ARJ/LAA ≤ 70%), or severe (ARJ/LAA > 70%) [32,33]. This method is affected by several technical and hemodynamic limitations [31], but has been demonstrated to correlate quite well to other more advanced Doppler techniques [33], and it is relatively easy to perform and not time-consuming. A more accurate method for quantifying MR is the proximal isovelocity surface area (PISA) [34]. Although this method is more time-consuming, it provides more information, allowing the calculation of effective regurgitant orifice area (EROA), regurgitant volume (RV), and regurgitant fraction (RF). However, even MR quantification based on PISA can lack accuracy or be precluded by eccentric or multiple regurgitant jets, and by orifice shapes different from circular [34,35]. Other methods that can be used for evaluating MR severity are the subjective assessment of the density of the continuous wave Doppler trace and its comparison to the intensity of the MV inflow signal [36,37], and the measurement of the diameter of the vena contracta [38].

#### 3.1.2. Assessing Cardiac Remodeling

Mitral regurgitation is often referred to cause a pure volume overload [39]. The extra volume that is added to the LA during systole by the MR re-enters the left ventricle (LV) at each diastole, increases the diastolic wall stress and triggers the response of atrial and ventricular remodeling. Given the progressive nature of LA and LV enlargement, serial measurements of these chambers are important steps for assessing disease severity and progression [40]. Moreover, LA and ventricular sizes, and their magnitude of change, proved to be important independent predictors of survival in a multitude of studies [23,24,29,30,41].

Left atrial dimensions can be quantified using several echocardiographic techniques [42,43,44]. The most common assessment of LA dimension entails standardization of the LA diameter to the animal body size through measurement of the aortic root diameter (Ao), and calculation of the ratio LA:Ao. In a mixed healthy canine population, this ratio is found to be <1.6 [42], and LA:Ao > 1.5 is commonly used to define LA enlargement [11,43,45]. However, given the complex shape and remodeling patterns of the LA, an assessment that takes into account different echocardiographic views [42], or a volumetric assessment of the LA [44,46], could constitute a more comprehensive evaluation of LA size. The bi-plane area-length method for determining LA volume performed superiorly to LA:Ao for identification of mild atrial enlargement in the study of Wesselowski et al. [44]. When evaluated with this technique, two studies proposed a maximum LA volume of 0.92 mL/Kg and 1.1 mL/Kg as normal reference values, respectively [44,46].

Assessing the LV also provides important information in the evaluation of disease severity and progression. The mechanisms involved in LV remodeling and enlargement secondary to MR are a set of complex mechanical stimuli and molecular responses [39], which lead to a progressive increase in end-diastolic and end-systolic volume. As for atrial dimensions, efforts have been made for producing estimates of LV dimensions that would faithfully reflect the progression in LV enlargement, and would be unrelated to the size of the dog [47,48,49,50]. One of the most accurate and extensive investigations of canine cardiac dimensions identified an allometric relationship between body weight and M-Mode derived LV end systolic diameter (LVIDs) and end diastolic diameter (LVIDd) [50], allowing the definition of prediction intervals for a wide range of body weights (BW). Briefly, the M-Mode derived LVIDd divided by the dog’s BW elevated to the power of 0.294 (BW^0.294^) should be ≤1.85, while the M-Mode derived LVIDs divided by the dog’s BW elevated to the power of 0.315 (BW^0.315^) should be ≤1.26; values greater than these will indicate LV enlargement. Systolic function could also be impaired in dogs with MMVD [3,4]. However, the evaluation of systolic function is difficult in dogs affected by MMVD because of the change in ventricular loading conditions. With the progression of the disease, in fact, MR imposes a progressive increase in preload and slight decrease in afterload. All the non-invasive indices of cardiac function are affected by these changes to some extent. Fractional shortening and ejection fraction are, in fact, usually increased in the hyper dynamic ventricle of an affected dog, and this reduces the sensitivity of these indices for assessing LV dysfunction [51]. In this condition, a decrease in systolic function could be suggested by an increase in end systolic left ventricular dimensions. Particularly, given the above-mentioned relationship between this measurement and body sizes, an allometrically scaled LVIDs > 1.26 indicates an increase in LVIDs, and can be suggestive of LV systolic dysfunction in patients affected by MMVD.

### 3.2. Advanced Echocardiographic Techniques

The continuous technological development of echocardiography keeps providing new, advanced tools for the evaluation of cardiac dimension and function. Tissue Doppler Imaging (TDI) allows quantification of myocardial motion, and its use in healthy dogs and dogs with MMVD for assessing the left and the right ventricles has been extensively investigated [52,53,54,55]. The most useful variable commonly obtained is the ratio between early diastolic mitral inflow peak velocity (E-wave) and the peak early diastolic motion of the parietal mitral annulus (e’-wave). With progression of MMVD, early LV filling is affected by increasing LA pressure and diastolic dysfunction, which have opposite impacts, making the assessment of these conditions difficult. Myocardial motion and, thus, the e’ wave, however, is less affected by loading conditions compared to the E-wave [56]. Therefore, the ratio E/e’ could be used for predicting LV filling pressures by removing most of the effect of the diastolic dysfunction [57]. However, these assumptions do not always hold in the severe volume overload caused by MMVD, or in scenarios in which diastolic function is preserved [58,59], limiting the ability of this variable to accurately predict LV filling pressures in dogs with severe MMVD.

Attempts have been made also to use tissue Doppler indices, as well as speckle-tracking-derived strain and strain rate, to obtain load-independent indices of LV systolic function [60,61]. However, from these studies a TDI or speckle-tracking derived index of systolic function for dogs with MMVD could not be identified.

Some TDI variables have also been reported to be useful to predict the presence of pulmonary arterial hypertension (PHT) in dogs affected by MMVD [55,62]. Particularly, in one study cutoff values of 10 and 9.33 for E/e’ ratio calculated using the e’ of the lateral and septal MV annulus, respectively, were moderately accurate in predicting the presence of PHT in dogs affected by MMVD [55]. In another study from Serres and colleagues, a global tricuspid TDI (defined as the parietal tricuspid annular systolic velocity multiplied by the ratio between early and late diastolic velocities) greater than 11.8 had 89% sensitivity and 93% specificity in distinguishing between normal dogs and dogs with an estimated systolic pulmonary arterial pressure >30 mmHg [62]. However, in these studies the pulmonary pressure was estimated on the basis of tricuspid regurgitant jet velocity, a technique known to lack accuracy [63,64].

The lack of a third dimension in conventional echocardiography poses a limit to the amount of information that can be derived from 2D images and cine-loops. Most of the calculation of areas and volumes that are performed using this technique rely on geometrical assumptions, attempting to approximate complex three-dimensional cardiac structures. Preliminary experiments in the development of three-dimensional echocardiography (3DE) were started during late 1970s and early 1980s [65,66,67,68] with the first dedicated analysis of the MV morphology in 1989 by Levine and colleagues [69]. Following decades of studies and validation against gold standards, transthoracic and transesophageal 3DE are now recommended diagnostics for evaluating cardiac chambers and valves in humans [70,71]. The first report of a 3D reconstruction of a dog’s ventricle using ultrasonographic data was published in 1979 [72]. However, it was 2010 before the first publication in the veterinary field regarding the use of real-time three-dimensional transthoracic echocardiography (RT-3DTTE) in clinical settings in dogs with and without acquired cardiovascular diseases was published [73]. In that study, conventional methods for estimating end-systolic and end-diastolic LV volumes (two 2D methods and the Teichholz method from M-Mode images) were compared to RT-3DTTE, finding good agreement between the 2D techniques and RT-3DTTE, and confirming a known overestimation of the LV volumes by the Teichholz method. In the same study, the estimation of LA sizes using RT-3DTTE was also investigated and compared to LA/Ao measures, finding increasing differences between the two techniques with atrial dilatation. Another study compared LA measurements obtained using RT-3DTTE in 32 healthy dogs to several 2D methods for estimating LA sizes [74]. In this study, only allometric scaling of 2D LA measurements correlated with LA volume obtained using RT-3DTTE [74].

Subsequently, an analysis of LV volumes in healthy dogs and dogs affected by MMVD was performed [75], and found that end-diastolic and end-systolic LV volumes significantly expand only in dogs that are severely affected, and that the central portion of the LV, compared to the basilar and apical portions, is the main contributor to this increase in volumes. Furthermore, the LV was found to change into a more spherical shape, with potential implications on the geometry of the MV apparatus.

**Figure 4:**
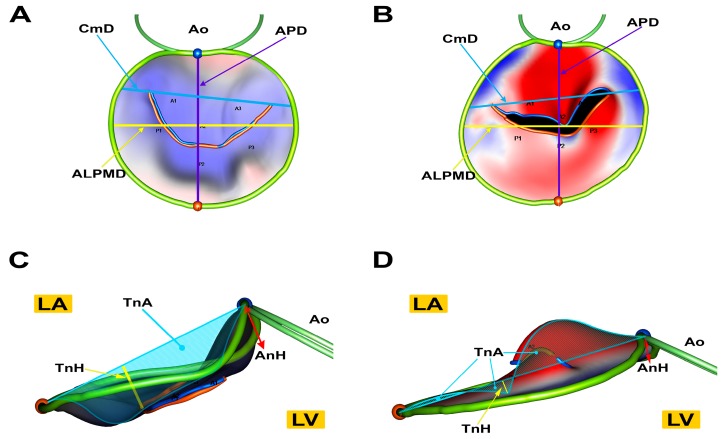
Atrial (**A**,**B**) and lateral (**C**,**D**) view of MV models of a healthy dog (**A**,**C**) and a dog affected by MMVD (**B**,**D**). Healthy dogs have a more elliptical annulus (**A**) while dogs with MMVD have a more circular one (**B**). Furthermore, in healthy dogs the saddle shape is more evident (**C**) than in dogs with MMVD (**D**) that have a smaller annulus height. Tenting height and tenting area are reduced in dogs with MMVD (**D**) compared to healthy dogs (**C**). Ao, aortic annulus; APD, antero-posterior annulus diameter; ALPMD, anterolateral- posteromedial annulus diameter; CmD, commissural diameter; LA, left atrial side of the valve; TnA, tenting area (dashed); AnH, annulus height; LV, left ventricular side of the valve; TnH, tenting height. Reproduced with permission from Menciotti et al., J. Vet. Cardiol.; published by Elsevier, 2017 [77].

In 2016, our group first reported the use of RT-3DTTE for performing a dedicated evaluation of the MV in healthy dogs [76]. In this study, we demonstrated that performing analysis of canine MVs using RT-3DTTE and a dedicated software for off-line analysis is feasible and repeatable. Additionally, we described how a population of healthy dogs of different breeds presents an overall elliptical, saddle-shaped MV annulus. Interestingly, in subsequent work, when healthy dogs were compared to dogs affected by MMVD, we found that dogs with the disease present an MV of abnormal morphology, which tends to lose its elliptical and saddle shape in favor of a more circular and flatter morphology [77] (Figure 4). The cross-sectional nature of this study, however, does not allow to infer whether the abnormal MV morphology is somehow a contributor to the etiopathogenesis of the disease, or just a consequence of the overall pathologic changes occurring in MMVD.

## 4. Therapy

### 4.1. Medical Therapy

Dogs affected by MMVD are mostly managed by administering drugs with the purpose of prolonging survival time. The “Guidelines for the diagnosis and treatment of canine chronic valvular heart disease” stipulated in 2009 by a panel of experts in veterinary cardiology, and currently undergoing a review process, defined the standard therapeutic guidelines for dogs affected by MMVD, along with the adoption of a staging system meant to guide the clinician in the therapeutic choices [26].

In general, dogs affected by hemodynamically-insignificant MMVD (without echocardiographic or radiographic evidence of cardiac enlargement, i.e., ACVIM Stage B1) are not suggested to receive any treatment, but it is advised to periodically monitor these dogs in order to identify disease progression.

In 2016, the conclusion of a multicenter, double-blind, placebo-controlled clinical trial investigating the efficacy of pimobendan for delaying the onset of CHF in dogs with preclinical MMVD (EPIC study) with LA and LV enlargement (i.e., ACVIM Stage B2) suggested that administration of pimobendan was associated with a prolongation of the preclinical period by approximately 15 months, compared to the placebo group [78]. Regarding the use of ACE-inhibitors in this stage of the disease, studies have shown contrasting results [79,80], and their administration is, therefore, not recommended by a consensus of the panelists. It is very important to remark, however, that ACVIM Stage B2 dogs represent a heterogeneous group of patients. Therefore, the benefit observed in the EPIC study can be inferred only for dogs that respect inclusion criteria of this study. In fact, for being enrolled in the aforementioned trial, dogs had to have evidence of significant left cardiac enlargement both at echocardiographic examination (LA/Ao ≥ 1.6, LVIDd divided by allometric scaled BW ≥ 1.7) and on thoracic radiographs (Vertebral Heart Score > 10.5).

Dogs affected by MMVD that develop CHF are considered to be in ACVIM Stage C. Chronic pharmacologic management of dogs in this stage is usually comprised of furosemide to provide diuresis, ACE-inhibitors and spironolactone to counteract renin-angiotensin-aldosterone system activation [80,81,82], and pimobendan to improve forward stroke volume [83,84]. However, Stage C dogs do require a tailored pharmacological approach in which drugs and their dosages are adjusted on the basis of the patient’s clinical condition. Management of the life-threatening, acute episodes of CHF or other complications, such as development of atrial fibrillation, may require the use of other drugs such as digoxin, diltiazem, hydralazine, and amlodipine. Since cough can be present, and may significantly affect quality of life, cough suppressants such as hydrocodone and bronchodilators are sometimes used [85,86].

When escalating the drugs’ dosages does not result anymore in the expected improvement in clinical conditions, the dog is considered in the refractory stage (i.e., ACVIM Stage D) of the disease. As for dogs in ACVIM Stage C, this category of patients requires a tailored approach that attempts to prolong survival mainly by means of diuresis, improvement of patient’s oxygenation status, reduction of afterload, control of arrhythmias and cough. Torsemide is a loop diuretic and chloride channel blocker which exerts a more potent and longer lasting diuresis than furosemide, produces less kaliuresis, and is also reported to have an aldosterone blocking effect [87,88,89,90]. The amount of scientific evidence relative to the safety and efficacy of this drug in dogs with MMVD and CHF is currently limited, and its use is usually reserved for dogs who become refractory to furosemide, i.e., dogs that experience reoccurrence of CHF despite high doses of furosemide (≥4 mg/kg/day) [88].

A potential complication associated with MMVD is the development of PHT as a result of elevated LV filling pressures [91,92]. Pulmonary hypertension has been recognized as a negative prognostic factor in dogs with MMVD, and an estimated systolic pulmonary pressure greater than 55 mmHg is an independent predictor of poor outcome in dogs with ACVIM Stage B2 and C MMVD [93]. Sildenafil is a selective phosphodiesterase-5 inhibitor which promotes pulmonary vasodilation, therefore, lowering the pulmonary pressure. Its use in dogs with PHT secondary to MMVD has not being extensively evaluated, but some studies identify a benefit in quality of life and clinical signs in dogs with PHT treated with sildenafil [94,95,96]. Therefore, its use in patients with CHF caused by MMVD and elevated pulmonary pressure may be considered. However, it is important to recognize that PHT in patients with left-sided heart failure is initially due to high LA pressure and that these patients usually respond well to diuretics. Therefore, it is opinion of the authors that in dogs with PHT and MMVD, sildenafil should be considered only in those cases when diuretics are not successful in lowering the pulmonary pressure.

### 4.2. Surgical Therapy

The treatment of choice in humans affected by severe MR is MV surgery [97]. The medical therapy mentioned in the previous paragraph, in fact, palliates the symptoms and prolongs survival of affected dogs, but has no effect on the main mechanism that leads to CHF, that is, MR due to the valvular lesions. Conventional MV repair techniques require cardio-pulmonary bypass in order to perform left atriotomy, direct visualization of the MV, mitral annuloplasty, and chordae tendinae replacement [98]. The final goal of the technique is to restore leaflet coaptation and, therefore, to diminish/abolish MR. Unfortunately, the technique requires a very well trained team and special equipment, which discourages the widespread use of this practice in veterinary medicine. To date in fact, routine successful MV repair in dogs is performed only by one team worldwide [98,99,100,101].

#### 4.2.1. Mitral Valve Replacement

Mitral valve replacement has been attempted in veterinary medicine. Attempts in replacing the canine MV have used mechanical valves and bioprosthetic valves. The first one represents an artificial implant, and the reports regarding this technique seem to describe very good results in the immediate post-operative period, but very poor long term prognosis mainly due to thrombosis [102,103]. Bioprosthetic valves instead use animal tissue as material in order to improve the biocompatibility of the implant. The few manuscripts reporting the use of these implants and long-term prognosis in dogs seem to provide encouraging results regarding prosthesis function, durability, and low thrombogenicity [104,105,106]. However, these implants require an open-heart surgery as well, and, therefore, suffer from most of the surgical limitations listed above. This is probably the most important factor preventing a more thorough investigation of these prostheses and therefore widespread use of them.

#### 4.2.2. Minimally-Invasive Approaches

In the last years, several devices had been developed for performing MV repair and replacement either through a transcatheter or a transapical approach. The main device which is currently used for transcatheter MV repair in humans is the MitraClip [107,108]. Delivered in the LA through a trans-septal approach, this device clips together two facing portions of anterior and posterior leaflet, therefore, performing an edge-to-edge MV repair. The device underwent two successful clinical trials and is currently Food and Drug Administration (FDA) and Conformité Européene (CE) approved for MV repair [107,109]. Transapical MV repair has also been recently introduced, and the main CE approved device for performing this procedure is currently the NeoChord DS-1000. Once introduced in the LV through a mini-thoracotomy, the device captures a prolapsing portion of a MV leaflet, and a pair of artificial chordae tendinae is sutured to the captured leaflet. The artificial chordae are then tensioned under transesophageal echocardiographic guidance and fixed to the LV apex [110]. Another similar device, also currently under investigation in human medicine with encouraging results, uses a LV thoracotomy and transapical approach to deliver expanded polytetrafluoroethylene (ePTFE) artificial cords to the prolapsing portions of the MV. The device is directed to the prolapsing portions of a leaflet under transesophageal guidance and, once actuated, it pierces the leaflet and anchors the ePTFE artificial cords on the atrial side of it through pre-formed knots. The ePTFE cord is then pulled out from the ventricular apex and its tension is adjusted under transesophageal echocardiographic guidance in order to reduce the prolapse [111]. Our group has recently tested this device in six healthy beagles demonstrating the technique’s feasibility, and early (30 days) endothelialization of the ePTFE implants [112]. Lastly, several devices for MV replacement through either transcatheter or transapical approach are at various stages of development and ongoing clinical trials [113]. All these techniques have the tremendous advantage of not requiring cardiopulmonary bypass to be performed and, therefore, would constitute a great asset for the therapy of MMVD in dogs. To the best of our knowledge, no scientific publication has so far reported the use of any of these techniques in veterinary medicine, but from personal experience and communications with other investigators, some veterinary centers are currently investigating the feasibility and efficacy of these devices in dogs.
